# Supplementary material for: FPGA acceleration of GWAS permutation testing
Source: Bioinform Adv. 2025 Jun 18;5(1):vbaf145. doi: 10.1093/bioadv/vbaf145 (PMC12237511; doi:10.1093/bioadv/vbaf145)
Supplement: vbaf145_Supplementary_Data [file vbaf145_supplementary_data.pdf]

Supplementary Material: FPGA Acceleration of GWAS Permutation Testing

Yaniv Swiel   Jean-Tristan Brandenburg   Mahtaab Hayat
Wenlong Carl Chen   Mitchell A. Cox
Scott Hazelhurst
University of the Witwatersrand,
1 Jan Smuts Avenue, 2001, Johannesburg

June 13, 2025

S1 Description of Data Set 1: General method of construction

Data set 1 is a synthetic data set designed to be used solely for training and computational benchmarking purposes. It captures allele frequency, local LD structure and some population structure based on African data.

Briefly it was constructed from real genotype data from 16,000 African individuals from two separate studies. The individuals come from Southern, East and West Africa. All individuals had been genotyped on the H3Africa Custom array, which was jointly designed by the Pan-African Bioinformatics Network for H3Africa and Illumina (`https://h3abionet.org/h3africa-chip`). Each individual is genotyped at ≈2m positions – Y-chromosome and mitochondrial data are ignored.

The individuals are clustered in 6 clusters with genetically similar individuals in each cluster. If any clusters have fewer than 100 individuals, smaller clusters are merged together so that the smallest cluster is at least 100 individuals in size.

The genome is divided up into ≈15,000 windows each with 128 SNPs (genetic markers).

We then process each cluster separately, generating enough new synthetic individuals per cluster in proportion to cluster size so that the total number of synthetic individuals is 2,100. For each synthetic individual, for each of the ≈15,000 windows, we randomly pick a corresponding window from a random real person. Where necessary, we make semi-random modifications to the X chromosome, picking a random 50% of the participants to be biologically male.

This process keeps the data faithful to the linkage disequilibrium structure and allele frequency spectrum. However, it flattens the population structure within each cluster since we average out the genetic diversity across the genome. The next step is to synthetically build in population structure:

- A principal component analysis of the original data is used to identify the most important SNPs contributing the principal components 1 and 2 of the population structure.
- We then semi-randomly distribute values across these SNPs to produce some structure in the cluster.

Phenotypes are randomly produced using the GTCA tool. We pick some fake condition and use GCTA to produce either case/control or quantitative data. The distribution of phenotype is random and is quite independent of the studies for which the original data was collected.

S2 Benchmarking choices

We compared our FPGA solution to our on premises solution (University Research Cluster) rather than a CPU-based AWS solution for several reasons.

First, we note that the performance of our FPGA system is at least an order of magnitude better than that of the CPU-based solution, and so even a factor of two difference in choice of CPU would not change our results.

Second, what is a fair comparison? For our FPGA solution there is only one choice of architecture and machine. However, with AWS CPU-based solutions there are dozens of instance types possible use and at least 5-10 which would be sensible to use but with different characteristics (architecture generation, memory, number of virtual cores, etc.) and so different performance and pricing. The advantage of using our cluster in this paper is that we used it and the FPGA-based solution in a real production run analysing data set 2. So the figures we present are based upon real production use rather than architectures cherry picked for performance purpose.

Third, the price of the AWS EC2 instances varies not only according to the instance but the time of day. We could quote the on demand price which is stable. However, a fair comparison requires the use of spot instances, the price of which varies depending on demand on AWS. Again this makes determining what a fair comparison is on AWS difficult.

Finally, there was a strong pragmatic reason – the cost. The computing costs of this project were not funded by any project and we had to use limited discretionary funding available for the FPGA work. We preferred to use our discretionary funding for e.g., student stipends.

We benchmarked our cluster nodes to two different AWS solutions, m5.8xlarge and m4.10xlarge as shown in Table A1. The estimated cost of the computation done on our machine if done on AWS is:

$$\text{Number of hours on local} \times \text{AWS/Local} \times \text{Spot Price}$$

Depending on which AWS architecture used and actual spot price, one hour of computation on our local machine would cost USD0.51–USD0.85 per hour. Note that the uncertainty on the cost is primarily due to the AWS solution used and actual pricing on the day or days, rather than the relative performance of our machines to AWS.

S3 Additional experimentation

We generated a new synthetic data set, i4s4, with 2.2m SNPs and 4461 individuals. Using PLINK's `--thin` and `--thin-indiv` options each with values 0.5 and 0.25 we produced sub-sampled data sets: i2s4 and i1s4 with approximately a half and a quarter the number of individuals as i4s4; and i4s2 and i4s1 with half and quarter the number of SNPs as i4s4. We ran this (performing 100 maxT permutations for each dataset) on our 40-core dual CPU Xeon Silver 4114 machine and on the f1.2xlarge FPGA instance, as shown in Table A2.

Type	CPU	Freq	Cores	Cache	Time	AWS/Local	Cost (USD)
m5.8xlarge	Platinum 8175M	2.5	32	33792	3676	0.81	0.63, 0.69
m4.10xlarge	E5-2676 v3	2.4	40	30720	4277	0.95	0.80, 0.90
Local	Silver 4114	2.2	40	14080	4515	–	–

Table A1: Benchmarking of local machine to two AWS solutions. *Type* – m5.8xlarge and m4.10xlarge are AWS instance types, Local is our cluster node; *CPU* – all CPUs were Intel Xeons as described; *Freq* is the frequency in Gigahertz; *Cores* – number of cores on the computer; *Cache* is the cache size in KB; *Times* is given in seconds; *AWS/Local* is the relative time of the AWS solution to the local machine (inverse of speed-up). *Cost* shows two per-hour spot prices for each AWS solution (29 November and 1 December 2023).

Scaling number of SNPs			Scaling number of individuals		
Data	CPU Run time (s)	FPGA Run time (s)	Data	CPU Run time (s)	FPGA Run time (s)
i4s1	2910	17	i1s4	3593	22
i4s2	5783	31	i2s4	6516	33
i4s4	11918	57	i4s4	11918	57

Table A2: Comparing the run time (in seconds) of FPGA-based maxT permutation to PLINK for different amounts of SNPs and individuals.
